# 253. Clinical Presentations of Hospitalized Adult Patients Infected with SARS-CoV-2 during the Omicron Variant Surge in South Florida

**DOI:** 10.1093/ofid/ofac492.331

**Published:** 2022-12-15

**Authors:** Jianli Niu, Myeongji Kim, Ayesha T Jalal, Jessica E Goldberg, Elsa M Acevedo Martinez, Nathalie P Suarez Moscoso, Heysu Rubio-Gomez, Daniel Mayer, Alvaro Visbal, Candice Sareli, Paula A Eckardt, Aharon E Sareli

**Affiliations:** Memorial Healthcare System, Hollywood, Florida; Memorial Healthcare System, Hollywood, Florida; Memorial Healthcare System, Hollywood, Florida; Memorial Healthcare System, Hollywood, Florida; Memorial Healthcare System, Hollywood, Florida; Memorial Healthcare System, Hollywood, Florida; Memorial Healthcare System, Hollywood, Florida; Memorial Healthcare System, Hollywood, Florida; Memorial Healthcare System, Hollywood, Florida; Memorial Healthcare System, Hollywood, Florida; Memorial Healthcare System, Hollywood, Florida; Memorial Healthcare System, Hollywood, Florida

## Abstract

**Background:**

Patients infected with COVID-19 Omicron variant, may be hospitalized for reasons other than COVID-19 pneumonia. We describe the clinical presentations of hospitalized adult patients with COVID-19 Omicron variant in a large healthcare system in South Florida.

**Methods:**

Laboratory-confirmed COVID-19 adult patients hospitalized during January 1-14, 2022 were retrospectively reviewed. Clinical presentations were divided into one of three admission groups: COVID-19 pneumonia or respiratory infection (Group 1), severe extrapulmonary manifestations of COVID-19 (Group 2), and completely incidental diagnosis of COVID-19 (Group 3). Risks of in-hospital mortality and intensive care admission were estimated using logistic regression models.

**Results:**

Among 500 consecutively hospitalized COVID-19 Omicron patients, the median age was 69 (IQR, 53-80) years, and 271 (54.2%) were women. The most common comorbidities were hypertension (326; 65.5%), diabetes (160; 32%), and chronic kidney disease (120; 24%). 260 (52%) patients were fully vaccinated (defined as a patient who received 2-dose vaccines), and 32 (6.4%) were previously infected with COVID-19. 257(51.4%) patients were classified as Group 1, 82 (16.4%) in Group 2, and 161 (32.2%) in Group 3 (Figure 1). Compared to Group 3, patients in Group 1 and Group 2 had a higher risk for ICU admission, with odds ratios (ORs) of 7.45 (95% CI, 2.62-21.23; p< 0.001) and 4.84 (95% CI, 1.44-16.23; p=0.011), and for in-hospital mortality, with ORs of 27.76 (95% CI, 3.78-204.3; p=0.001) and 12.63 (95% CI, 1.49-106.78; p=0.020), respectively (Figure 2). In multivariable-adjusted models, patients in Group 1 remained at higher risk for ICU admission and in-hospital mortality compared to Group 3, while patients in Group 2 remained at a higher risk for ICU admission, but with no difference in in-hospital mortality compared to Group 3 (Figure 2).

**Figure 1.**
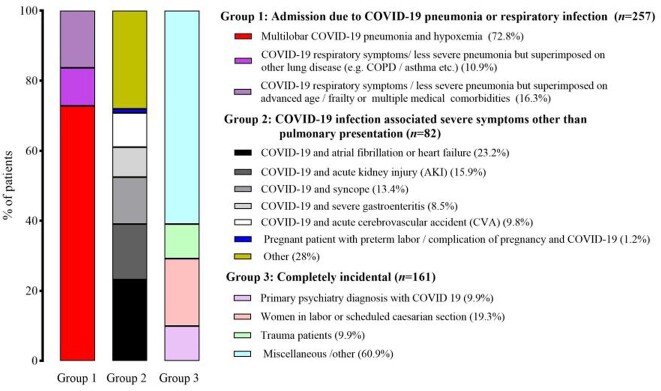
Clinical characteristics of consecutively hospitalized patients stratified by clinical presentations at admission.

**Figure 2.**
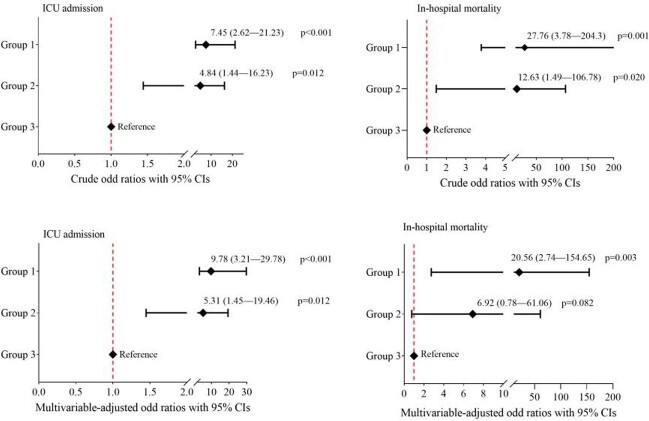
Crude (Upper panel) and multivariable-adjusted (Lower panel) odds ratios for ICU admission and in-hospital mortality from logistic regression models. Group 3 represents patients with a completely incidental diagnosis of COVID-19. The variables included in the final multivariable models were age, gender, history of hypertension, diabetes, chronic obstructive pulmonary disease, chronic kidney disease, coronary artery disease, malignancy, transplantation, HIV, vaccination status, and previous SARS-CoV-2 infection.

**Conclusion:**

This case series illustrates the clinical presentations of hospitalized adult patients infected with the COVID-19 Omicron variant. Significant differences in in-hospital mortality and ICU admission exist when comparing patients admitted for a COVID-19 related respiratory infection to those admitted with a completely incidental COVID-19 diagnosis.

**Disclosures:**

**All Authors**: No reported disclosures.

